# Machine Learning Models for Predicting Mortality in 7472 Very Low Birth Weight Infants Using Data from a Nationwide Neonatal Network

**DOI:** 10.3390/diagnostics12030625

**Published:** 2022-03-03

**Authors:** Hyun Jeong Do, Kyoung Min Moon, Hyun-Seung Jin

**Affiliations:** 1 Department of Pediatrics, Gangneung Asan Hospital, University of Ulsan College of Medicine, 38, Bangdong-gil, Sacheon-myeon, Gangneung-si 25440, Korea; firely1@hanmail.net; 2 Department of Pulmonary, Allergy and Critical Care Medicine, Gangneung Asan Hospital, University of Ulsan College of Medicine, 38, Bangdong-gil, Sacheon-myeon, Gangneung-si 25440, Korea

**Keywords:** machine learning, mortality, prediction, premature birth, infant, newborn

## Abstract

Statistical and analytical methods using artificial intelligence approaches such as machine learning (ML) are increasingly being applied to the field of pediatrics, particularly to neonatology. This study compared the representative ML analysis and the logistic regression (LR), which is a traditional statistical analysis method, using them to predict mortality of very low birth weight infants (VLBWI). We included 7472 VLBWI data from a nationwide Korean neonatal network. Eleven predictor variables (neonatal factors: male sex, gestational age, 5 min Apgar scores, body temperature, and resuscitation at birth; maternal factors: diabetes mellitus, hypertension, chorioamnionitis, premature rupture of membranes, antenatal steroid, and cesarean delivery) were selected based on clinical impact and statistical analysis. We compared the predicted mortality between ML methods—such as artificial neural network (ANN), random forest (RF), and support vector machine (SVM)—and LR with a randomly selected training set (80%) and a test set (20%). The model performances of area under the receiver operating curve (95% confidence interval) equaled LR 0.841 (0.811–0.872), ANN 0.845 (0.815–0.875), and RF 0.826 (0.795–0.858). The exception was SVM 0.631 (0.578–0.683). No statistically significant differences were observed between the performance of LR, ANN, and RF (i.e., *p* > 0.05). However, the SVM model was lower (*p* < 0.01). We suggest that VLBWI mortality prediction using ML methods would yield the same prediction rate as the traditional statistical LR method and may be suitable for predicting mortality. However, low prediction rates are observed in certain ML methods; hence, further research is needed on these limitations and selecting an appropriate method.

## 1. Introduction

For treating critically ill newborns, the clinical situation or severity at the time of hospitalization often affects newborns’ subsequent death or morbidity [1,2]. Therefore, we can use it to evaluate the severity of the infant’s condition and predict the infant’s mortality outcome by combining medical history, clinical symptoms, and various test results obtained at the time of admission. The gestational age (GA) and birth weight (BW) of newborns affect prognosis; however, various neonatal severity assessment tools consider multiple conditions at the time of hospitalization [1,2,3,4,5]. In addition, owing to the significant influence of GA and BW, some studies have shown better prediction rates using these metrics alone [6].

Neonatal mortality or criticality assessments typically use general linear models, such as logistic regression (LR) analysis, to compare the influence of each risk factor; these models use prominent, influential factors as elements of assessment tools. However, LR models require statistical assumptions, such as linear relationships between the covariates and the risk of morbidity. Furthermore, limitations of regression, such as overfitting and multicollinearity, exclude the analysis of many explanatory variables. These limitations impede analytical models that select a small set of variables relevant to the clinical model [7].

Recently, ML techniques have emerged as the most popular and flourishing discipline in artificial intelligence (AI) due to the capabilities of high-performance hardware such as graphic processing units and the availability of large datasets. ML techniques have been applied to many medical disciplines, including in the detection of specific clinical findings in medical images, and have achieved excellent performance with high sensitivity and specificity [8,9]. Existing studies have used ML techniques to predict specific morbidity or in-hospital mortality; some studies reported better performance and lower error rates in predicting clinical outcomes compared to those obtained via logistic or Cox regression [6,8,9]. In neonatology, the number of studies using such ML technology or AI is increasing [10,11,12,13,14]. Some studies have analyzed neonatal mortality [15,16,17,18], according to a recent systemic review by Mangold et al., which analyzed 11 studies on ML technology and neonatal mortality [19]. Each study has pros and cons, such as sample size, time of data collection, handling of missing values, and type of prediction.

This study aims to determine the best model for mortality prediction by comparing the predictive performances of LR, artificial neural network (ANN), random forest (RF), and support vector machine (SVM) models using data from the Korea Neonatal Network (KNN), which is a national-wide registry of newborns weighing less than 1500 g who are admitted to the neonatal intensive care unit (NICU).

## 2. Materials and Methods

### 2.1. Study Design and Population

We conducted the ML analysis of the KNN data [20]. The KNN is a nationwide web-based registry for very low birth weight infants (VLBWI) in Korea that has been in operation since 2013. Approximately 70 hospitals participate, and its data account for more than 70% of the total admissions of VLBWI born in Korea [20,21].

**Figure 1:**
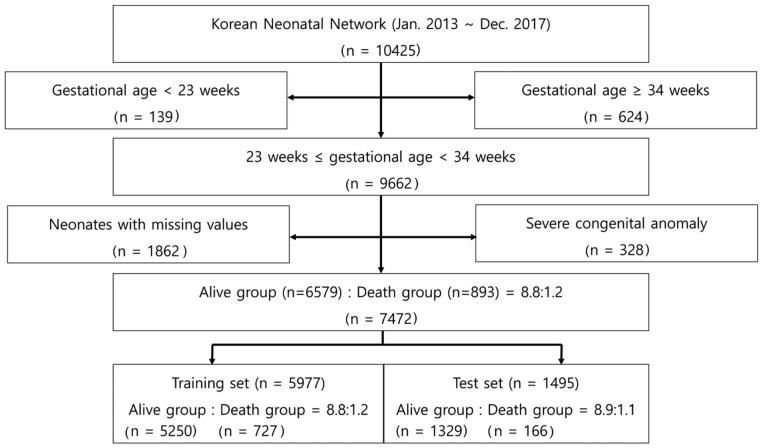
Flowchart outlining study methodology.

Using a standard electronic report form, we collected the data for 10,425 VLBWI born between 1 January 2013 and 31 December 2017 in 67 hospitals participating in the KNN. This study excluded 139 infants born at a GA of ˂23 weeks, 624 infants born at GA of ≥34 weeks (Figure 1 and Figure S1A,B), 328 infants with severe congenital anomalies, and 1862 infants who contained missing values (Figure 1). Thus, we included 7472 infants with complete records. The ratio of living infants (6579 infants) to dead infants (893 infants) was 8.8:1.2. For the ML analysis, we divided the dataset into a training set and a test set in a ratio of 8 (5977 infants):2 (1495 infants), and the ratios of living to dead infants for the training and test sets were 8.8 (5250 infants):1.2 (727 infants) and 8.9 (1329 infants):1.1 (166 infants), respectively (Figure 1). The data for each set were randomly selected.

### 2.2. Study Variables

The primary outcome of this study was obtaining the mortality prediction of VLBWI in the NICU. We analyzed 20 variables from the KNN registry (neonatal factors: male sex, GA, BW, 1 min and 5 min Apgar scores, body temperature, multiple births, and resuscitation at birth; maternal factors: age, diabetes mellitus (DM), hypertension, chorioamnionitis, premature rupture of membranes (PROM), antenatal steroid, and cesarean delivery; maternal social history: education, marital status, and nationality; paternal social history: education, and ethnicity). BW and GA showed a strong positive correlation of 0.73. Owing to the high possibility of multicollinearity between these two factors, the authors retained GA for the analysis (because it is considered a more clinically crucial factor) and excluded BW from the analysis (Supplementary Figure S2A). Additionally, because the evaluation items of the 1 min and 5 min Apgar scores are similar, the possibility of multicollinearity is high. The authors employed the 5 min Apgar score, which is considered more clinically crucial, in this study and excluded the 1 min Apgar score from the analysis. We excluded maternal and paternal social histories, including the highest education level, marital status, and nationality (Supplementary Table S1A,B). Finally, we analyzed the ML models using 11 predictor variables (neonatal factors: male sex, GA, 5 min Apgar scores, body temperature, and resuscitation at birth; maternal factors: DM, hypertension, chorioamnionitis, PROM, antenatal steroid, and cesarean delivery) (Supplementary Table S2 and Figure S2B).

### 2.3. Computational and Statistical Analysis

We randomly selected 80% of the total cases as the dataset for training mortality prediction by developing four different ML models—LR, ANN, RF, and SVM. We then selected the remaining 20% of cases as the test dataset to predict mortality. The four ML models considered are briefly reviewed below:

- LR is a powerful model to analyzing multiple explanatory variables simultaneously while reducing the impact of confounding factors [22]. We performed LR analysis to identify independent predictors used in the development of a multivariable prediction model. To avoid multicollinearity, we excluded variables with high correlation before the examination. We performed backward stepwise variable selection using a cutoff of *p* < 0.10.
- ANN is an ML model that consists of three layers containing connected nodes: an input layer, a hidden layer, and an output layer [23]. During training, the ANN adjusts the weights to learn how to predict the output. Each input variable appears as a node. The hidden layer contains multiple nodes determined during the model tuning phase. The output layer contains several nodes; the number of nodes equals the number of classes to predict. There is a weighted link between these layers, and the hidden layer receives the sum of the product of the associated weight value and the input variable plus the bias. This value is entered into an activation function to determine the class production. We used Tensorflow (available at http://www.tensorflow.org, accessed on 30 December 2021), an open-source ML library from the Google Barin Team, and an R interface to Keras (available at http://keras.rstudio.com, accessed on 30 December 2021).
- RF is an ensemble technique that improves generalization by combining multiple learning models [17]. The rationale behind an ensemble approach is that a pool of simple models may provide better performance than overfitted models because of significant variance. RF realizes ensembles of decision trees. Each tree describes the decisional process so that a branch decision is made by comparing the value of one feature at each node to a threshold. The tree’s structure and the thresholds are determined during the learning phase. RF builds multiple decision trees trained on training samples and combines the predictions to produce an ensemble output.
- SVM is an ML model based on a linear delimiter, suitable for binary classification. We extend SVM to handle non-linear problems using the so-called kernel tricks. It implicitly maps the input vectors into a high-dimensional feature space via a non-linear map derived via a kernel function [24]. SVM is one of the most popular learning algorithms and neural networks with excellent predictive accuracy and generalization capabilities. However, in large-scale problems, the computational complexity becomes very high, and the learning results’ interpretability is often difficult.

In this study, correlation between neonatal and maternal parameters and the mortality of VLBWI in NICU were analyzed using the Chi-squared test. We used the Student’s *t*-test to examine the differences among continuous variables. A two-tailed *p*-value of < 0.05 was considered statistically significant. Receiver operating characteristic (ROC) curves were used to plot the trade-off between specificity and sensitivity. The measures for the prediction performance of the ML model were: area under the ROC curve (AUROC), accuracy, sensitivity, specificity, positive predictive value (PPV), and negative predictive value (NPV). All data were cleaned and subsequently analyzed using R software packages (v.4.0.2; www.r-project.org, accessed on 30 December 2021).

## 3. Results

### 3.1. Demographic Characteristics of Patients

A total of 10,425 VLBWI were registered during the study period: 2953 neonates were excluded from this study because of considerations including GA, severe congenital anomalies, or missing values in their data. Thus, the final analysis included 7472 patients with complete records (Figure 1 and Figure S1A,B). Among the 7472 neonates, the “living group” size was 6579, and the “dead group” size was 893. Thus, the proportion of dead neonates in this study was 11.95% (Table 1).

### 3.2. Neonatal Clinical Characteristics of Living and Dead Groups

Patient characteristics are shown in Table 1. There were statistically significant differences between the two groups in sex, GA, birth weight, 1 min Apgar score, 5 min Apgar score, body temperature at admission, and resuscitation at birth (male sex: *p* = 0.035; others: *p* < 0.001). The mean GA at birth was 28^+4^ weeks (28^+6^ weeks in the living group vs. 25^+6^ weeks in the dead group), and the mean birth weight was 1071.0 g (1111.1 g in the living group vs. 775.2 g in the dead group). Moreover, 1 min Apgar score, 5 min Apgar score, and body temperature at admission were lower in the dead group than the living group (4.8 in the living group vs. 3.2 in the dead group, 7.0 in the living group vs. 5.5 in the dead group, 36.2 in the living group vs. 35.8 in the dead group, respectively). In addition, the dead group had a higher rate of resuscitation at birth than the living group (5914 (89.9%) in the living group vs. 881 (98.7%) in the dead group). However, there was no statistically significant difference between the two groups in multiple births (*p* = 0.367) (Table 1).

### 3.3. Maternal Clinical Characteristics of Living and Dead Groups

The average maternal age was 33 years; no statistically significant difference in this metric was observed between the two groups (33 in the living group vs. 33 in the dead group) (Table 1). However, statistically significant differences between the two groups were revealed in terms of maternal DM, hypertension, histologic chorioamnionitis, premature rupture of membrane (PROM), antenatal steroids, and the delivery mode (*p* < 0.001 in all groups). The maternal DM, hypertension, and antenatal steroids were higher in the living group than in the dead group (9.6% in the living group vs. 5.9% in the dead group; 22.1% in the living group vs. 15.6% in the dead group; 84.2% in the living group vs. 79.6% in the dead group, respectively). However, histologic chorioamnionitis and PROM rates were higher in the dead group than in the living group (35% in the Alive group vs. 41.9% in the dead group; and 36.3% in the living group vs. 41.4% in the dead group) (Table 1). In addition, a statistically significant difference in maternal education was observed between the two groups (*p* = 0.008). The rate of “college or above” maternal education was higher in the living group (74.4%) than in the dead group (71.8%) (Supplementary Table S1A). No statistically significant differences were observed between the two groups in terms of maternal marriage, paternal education level, or parents’ ethnicities (Supplementary Table S1B). 

### 3.4. Comparison of Prediction Performance of ML Models

**Figure 2:**
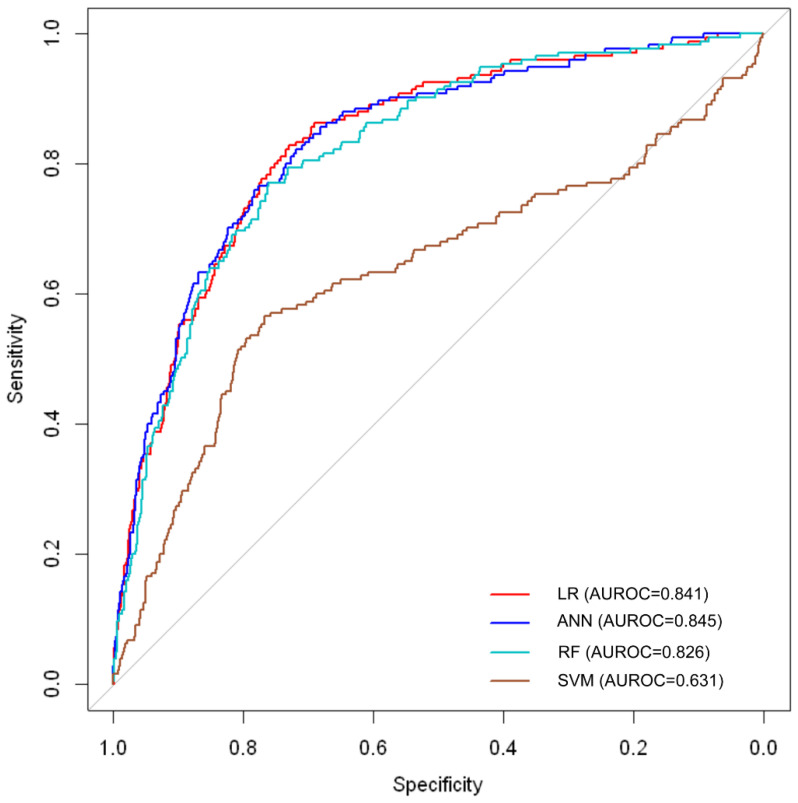
The receiver operating characteristic curve shows the statistical performance of mortality prediction by the LR, ANN, RF, and SVM models in very low birth weight infants. LR = logistic regression; ANN = artificial neural network; RF = random forest; SVM = support vector machine.

Table 2 and Figure 2 reveal the differential performances of ML models in predicting neonatal mortality. The AUROC (95% CI) of LR, ANN, RF, and SVM models were 0.841 (0.811–0.872), 0.845 (0.815–0.875), 0.826 (0.795–0.858), and 0.631 (0.578–0.683), respectively. The ANN yielded a higher AUROC (0.845), specificity (0.780), and NPV (0.964) than the LR, RF, and SVM models. The LR model yielded a higher accuracy (0.889), sensitivity (0.977), and PPV (0.905) than the ANN, RF, and SVM models. No statistically significant differences were observed between LR, ANN, and RF models in terms of the AUROC (Reference, *p* = 0.858, and *p* = 0.118, respectively). However, a statistically significant difference was observed between LR and SVM in terms of the AUROC (Reference and *p* < 0.001). The AUROC indicating the mortality prediction of the SVM model was lower than those of the LR, ANN, and RF models (Table 2 and Figure 2).

## 4. Discussion

The most notable result of this study is that a ML approach toward predicting neonatal mortality in VLBWI achieved similar performance power in ANN and RF, but not SVM, compared to LR. Several studies have reported that ML methods achieve better performance than LR in predicting neonatal mortality [16,17,18,25]. Compared with these studies, other ML models do not show better predictive power than LR but show similar performance power in this study. However, there are important differences between previous studies and this study. Previous studies compared predictions of neonatal mortality between the ML methods and traditional LR, while we used the ML method, AI-based LR in this study.

The problem of predicting the mortality of premature infants has a long history. The risk of neonatal death is mainly determined by the immaturity of organs and functions, which are indicated via low GA and BW values. Previous studies have identified GA and BW as the most critical risk factors affecting neonatal mortality [10,26,27,28]. However, since GA and BW are independent factors affecting VLBWI death, we believed using GA and BW together during the analysis would increase the risk of bias due to multicollinearity. Therefore, to reduce the multicollinearity error, we used a correlation matrix to understand the correlation between the two variables, wherein GA and BW showed a value of 0.73. This implies that the two variables have a strong linear relationship; therefore, there is a high risk of bias and the possibility of inaccuracy in the interpretation of the results. Therefore, of the two variables, we selected and analyzed the GA to predict neonatal mortality in VLBWI.

In addition, studies using ML may face a high risk of bias owing to limited reporting of sample size data, management of missing data, reporting of model results, and explanations or adjustments for missing data. In previous studies focused on predicting neonatal and perinatal mortality using ML methods, researchers tried to reduce the risk of bias and improve the predictive accuracy [10,16,17,29,30]. In particular, controlling missing values and the selection of variables seems to create a significant impact in ML studies. Ambalanan et al. [16] eliminated incomplete cases, and Podda et al. [17] and Shukla et al. [31] excluded infants with missing data to reduce the risk of bias due to missing values. In addition, Liu et al. [32] tried to reduce the risk of bias due to missing values by performing simple imputation of missing data. In our study, we removed the missing values (without processing) and incomplete cases to reduce the risk of bias due to missing values. In addition, to increase the accuracy of the predictive power of the ML models, various variables that can affect neonatal and perinatal mortality have been used in ML studies. Sheikhtaheri et al. [10] used 17 variables to predict neonatal mortality, and Cooper et al. [29] used 68 variables to predict neonatal mortality using a super learning algorithm. Mboya et al. [30] used 32 predictive variables to predict perinatal mortality. In this study, we use 20 variables during the initial admission state from KNN data for data analysis (Table 1 and Table S1A,B). GA was selected from among BW and GA at birth to reduce the risk of bias due to multicollinearity. Finally, we selected 11 variables (neonatal factors: male sex, GA, 5 min Apgar scores, body temperature, and resuscitation at birth; maternal factors: DM, hypertension, chorioamnionitis, PROM, antenatal steroid, and cesarean delivery) via statistical analysis and consideration of clinical impact without missing values for analysis in this study. All the variables were used by each model, and the prediction rates were compared afterward.

We applied four ML models (ANN, RF, SVM, and LR) to predict neonatal mortality in VLBWI. We found no statistically significant differences in the predictive performance of the ANN and RF models upon comparison with the LR, except for SVM, which had lower predictive performance. The ANN and RF models showed a predictive performance similar to LR; however, the sensitivity and specificity of each ML model differed. The RF model had sensitivity and specificity similar to those of LR, and the ANN model had a higher specificity but lower sensitivity than those of the LR model. As a result of comparing the prediction performance of the four models developed in this study, it showed that traditional LR method is close to ANN and RF, much better than SVM. SVM has the advantage of being well-suited for complex classification [24]. However, in this study, the variable importance of GA is very significant, and it is considered to be a classification that is not very complex, resulting in such a result. From the results of this study, marginal differences were observed depending on the type of ML method, importance of data and variable selection used for analysis, and appropriateness of the selected ML method. We analyzed the data using XGBoost (Extreme Gradient Boosting); the accuracy was the lowest at 0.377 (not shown). In addition, we performed analysis using 5- and 10-fold cross-validation. Both 5- and 10-fold cross-validation accuracy were lower than the four ML models as 0.606 (not shown).

LR is the most common algorithm for prognostic modeling, and it is often compared to AI-based models [33]. However, this should not be confused with the LR ML approach. Unlike our study, which compared AI-based LR with other ML models, several studies compared the traditional LR and ML approach. Several studies have reported that the ML approach provides better predictions of neonatal mortality than LR [16,17,18,25]. Conversely, other studies have reported that LR yields a better predictive power in the case of neonatal mortality than the ML approach [15,31,32]. Although it is not clear why the results of these studies differed, the studied samples differed in several important variables, such as GA range, BW, and presence or absence of congenital abnormalities. Therefore, we speculated the reason for different results from each previous study as these datasets influence the different variables. Additionally, the variables used in each study are partially different.

The ML approach facilitates a more refined approach to predicting the mortality of the preterm infants than that offered by the LR; thus, it can yield unbiased results and exhibit high clinical applicability. The ML approach allows the combination of higher-order non-linear correlation between the predictors, offers the advantage of being a non-parametric approach, and does not use distribution assumptions. The use of cross-validation also minimizes the possibility of overfitting in ML models. Therefore, the clinical application of ML models can help predict VLBWI mortality, and it can help make treatment decisions for this patient population. Although our study does not show results consistent with previous studies suggesting that either traditional LR or ML are superior, it does show that the ANN and RF models, but not SVM, can be as valuable as LR in predicting the mortality of VLBWI. Furthermore, the ANN and RF models showed a similar predictive ability of mortality in VLBWI compared to LR in this study. The LR method used to predict neonatal mortality in this study is considered to show similar predictive ability in ANN, RF, and LR, including the AI-based LR method which was used instead of the traditional LR method.

The first strength of this study is that we use a population-based, prospectively collected national cohort database that covered more than 70% of the total VLBWI in Korea and registered multi-center clinical information on VLBWI from the 60–70 participating hospitals in Korea. The second strength of this study is that we confirmed the correlation between two variables, GA and BW, that can independently affect mortality in VLBWI and lead to bias, using the correlation meter to minimize bias. Finally, the third strength of this study is that we removed missing values (without processing) and incomplete cases to reduce the risk of bias due to missing values. Therefore, the risk of bias due to missing values was low.

There are some limitations of this study that should be acknowledged. First, we excluded observations with missing values, which may be a disadvantage leading to an underestimating VLBWI mortality in this study. Second, although many variables may affect VLBWI mortality, we selected 11 variables for the analysis, which may lead to a selection bias in this study.

## 5. Conclusions

This study shows that when ML models are applied to predict VLBWI mortality using maternal and neonatal factors, the ANN and RF models, but not SVM, show a predictive power similar to LR. Moreover, the RF model shows sensitivity, specificity, and accuracy similar to the LR ML model. Therefore, we thought these ML models would be a valuable and alternative strategy to LR when predicting VLBWI mortality. However, there is a low prediction rate depending on the ML method, so further research is needed based on these limitations and selecting an appropriate method.

## Figures and Tables

**Table 1:** Clinical characteristics of the living and dead groups of KNN dataset.

Clinical Manifestation			Total (*n* = 7472)	Living (*n* = 6579)	Dead (*n* = 893)	*p*-Value
Neonatal factors	Sex (male), *n* (%)		3794 (50.8)	3311 (50.3)	483 (54.1)	0.035 ^1^
	GA		28 weeks 4 days	28 weeks 6 days	25 weeks 6 days	<0.001 ^2^
	BW, (g)		1071.0	1111.1	775.2	<0.001 ^2^
	Apgar Scores (1 min)		4.7	4.8	3.2	<0.001 ^2^
	Apgar Scores (5 min)		6.8	7.0	5.5	<0.001 ^2^
	Body temperature at admission, ℃		36.1	36.2	35.8	<0.001 ^2^
	Multiple birth, *n* (%)		2552 (34.2)	2259 (34.3)	293 (32.8)	0.367 ^1^
	Resuscitation at birth, *n* (%)		6795 (90.9)	5914 (89.9)	881 (98.7)	<0.001 ^1^
Maternal factors	Age, year, median (range)		33.0 (13–47)	33.0 (13–47)	33.0 (17–49)	
	DM, *n* (%)		687 (9.2)	634 (9.6)	53 (5.9)	<0.001 ^1^
	Hypertension, *n* (%)		1593 (21.3)	1454 (22.1)	139 (15.6)	<0.001 ^1^
	Histologic chorioamnionitis, *n* (%)		2679 (35.9)	2305 (35.0)	374 (41.9)	<0.001 ^1^
	PROM, *n* (%)		2759 (36.9)	2389 (36.3)	370 (41.4)	<0.001 ^1^
	Antenatal steroid, *n* (%)		6249 (83.6)	5538 (84.2)	711 (79.6)	<0.001 ^1^
	Delivery mode, *n* (%)	Vaginal	1652 (22.1)	1414 (21.5)	238 (26.7)	<0.001 ^1^
		Cesarean	5820 (77.9)	5165 (78.5)	655 (73.3)	

^1^ = Chi-squared test; ^2^ = Student *t*-test.

**Table 2:** Prediction performance of ML models on the test set.

Model	Logistic Regression	Artificial Neural Network	Random Forest	Support Vector Machine
AUROC (95% CI)	0.841 (0.811–0.872)	0.845 (0.815–0.875)	0.826 (0.795–0.858)	0.631 (0.578–0.683)
Accuracy	0.889	0.778	0.884	0.842
Sensitivity	0.977	0.765	0.976	0.921
Specificity	0.205	0.780	0.170	0.228
PPV	0.905	0.302	0.901	0.902
NPV	0.539	0.964	0.476	0.271
*p*-value *	Reference	0.858	0.118	<0.001

* We calculated *p*-values to compare the AUROC of logistic regression with each ML model; AUROC = area under the receiver operating curve; NPV = negative predictive value; PPV = positive predictive value.

## Supplementary Materials

The following are available online at https://www.mdpi.com/article/10.3390/diagnostics12030625/s1, Figure S1A: Scatter plots between gestational age and body weight at birth (100 g) for each admission year between 2013 to 2017, Figure S1B: Scatter plots between gestational age and a body weight at birth (100 g) based on each alive/death infant, Figure S2A: The correlation heatmap between features of this study and mortality. mSex = male sex; GA = gestational age; BW = body weight; Apgar = 5 min Apgar score; BT = body temperature; Resu = resuscitation; mDM = maternal diabetes mellitus; mHTN = maternal hypertension; Chor = chorioamnionitis; PROM = premature rupture of membranes; aStrd = antenatal steroid; cesar = cesarean delivery, Figure S2B: Correlation heatmap between features of this study and mortality. mSex = male sex; GA = gestational age; Apgar = 5 min Apgar score; BT = body temperature; Resu = resuscitation; mDM = maternal diabetes mellitus; mHTN = maternal hypertension; Chor = chorioamnionitis; PROM = premature rupture of membranes; aStrd = antenatal steroid; cesar = cesarean delivery, Table S1A: Maternal social history of the living and dead groups, Table S1B: Paternal social history of the living and dead groups. Table S2: Comparison of variables between KNN registry and this study.
