# Supplementary material for: Machine Learning Models for Predicting Mortality in 7472 Very Low Birth Weight Infants Using Data from a Nationwide Neonatal Network
Source: Diagnostics (Basel). 2022 Mar 3;12(3):625. doi: 10.3390/diagnostics12030625 (PMC8947011; doi:10.3390/diagnostics12030625)
Supplement: Supplementary file 1 [file diagnostics-12-00625-s001.zip › Supplementary Figures S1 and S2.pdf]

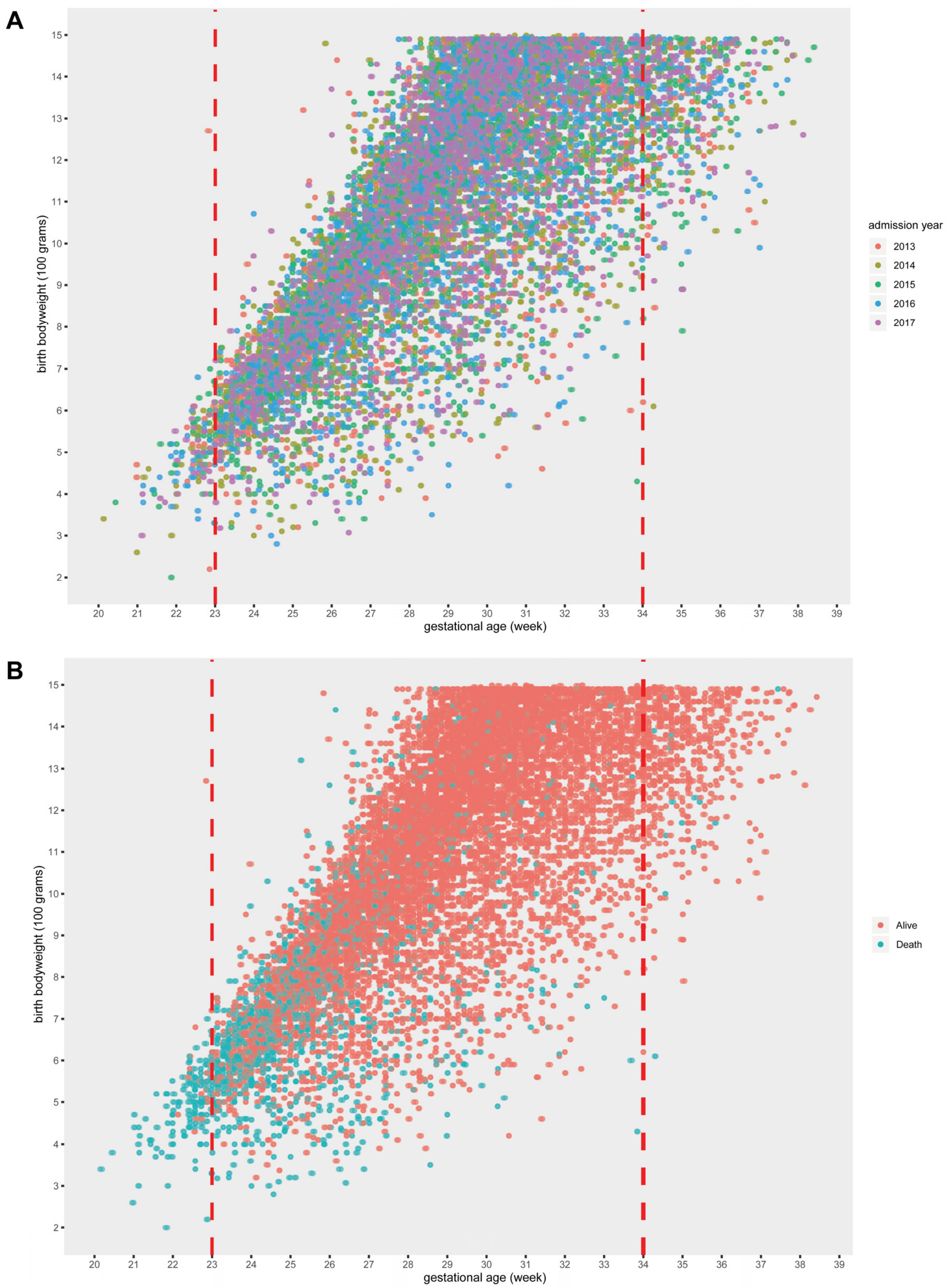

Figure S1. (**A**) Scatter plots between gestational age and body weight at birth (100 g) for each admission year between 2013 to 2017, (**B**) Scatter plots between gestational age and a body weight at birth (100 g) based on each alive/death infant.

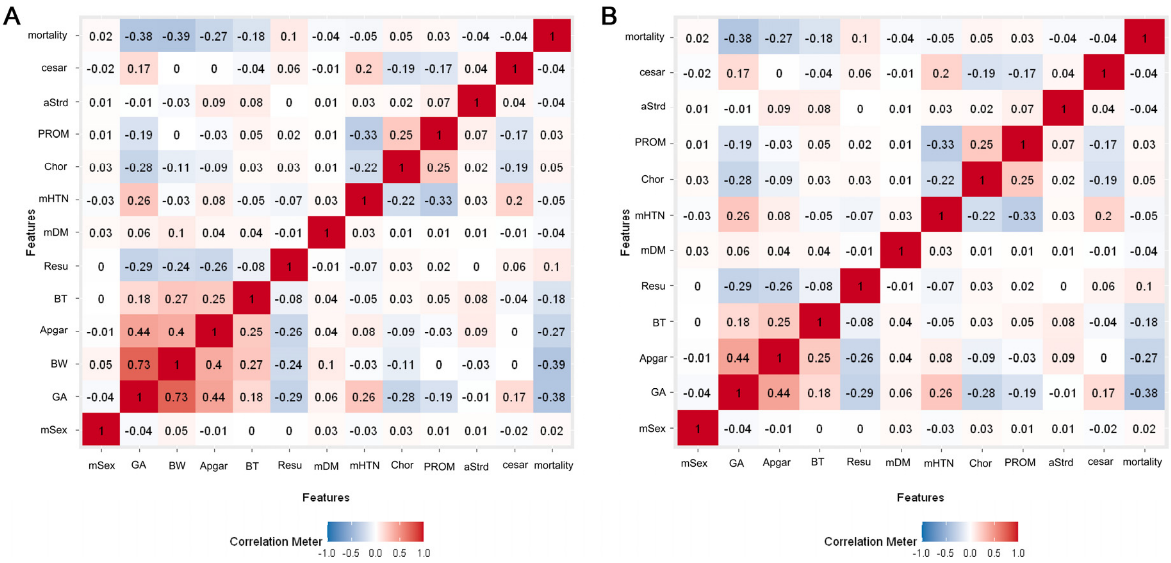

Figure S2. (**A**) The correlation heatmap between features of this study and mortality. mSex = male sex; GA = gestational age; BW = body weight; Apgar = 5 min Apgar score; BT = body temperature; Resu = resuscitation; mDM = maternal diabetes mellitus; mHTN = maternal hypertension; Chor = chorioamnionitis; PROM = premature rupture of membranes; aStrd = antenatal steroid; cesar = cesarean delivery, (**B**) Correlation heatmap between features of this study and mortality. mSex = male sex; GA = gestational age; Apgar = 5 min Apgar score; BT = body temperature; Resu = resuscitation; mDM = maternal diabetes mellitus; mHTN = maternal hypertension; Chor = chorioamnionitis; PROM = premature rupture of membranes; aStrd = antenatal steroid; cesar = cesarean delivery.
