# Supplementary material for: Machine Learning Models for Predicting Mortality in 7472 Very Low Birth Weight Infants Using Data from a Nationwide Neonatal Network
Source: Diagnostics (Basel). 2022 Mar 3;12(3):625. doi: 10.3390/diagnostics12030625 (PMC8947011; doi:10.3390/diagnostics12030625)
Supplement: Supplementary file 1 [file diagnostics-12-00625-s001.zip › SupplementaryT_1A.pdf]

Supplementary Table S1A. Maternal social history of the Alive and the Death groups

Characteristics	Alive (n=8127)	Death (n=1207)	*P*-value
**Social History**			
Education, graduation, *n* (%)			**0.008**[1]
College or above	4688 (74.4)	671 (71.8)	
High school	1504 (23.9)	235 (25.1)	
Middle school	82 (1.3)	25 (2.7)	
Elementary school	23 (0.4)	4 (0.4)	
Marital status			0.136[1]
Married	7967 (98.0)	1171 (97.0)	
Divorced	7 (0.1)	1 (0.1)	
Single	68 (0.8)	15 (1.2)	
Single living together	85 (1.0)	20 (1.7)	
Nationality, *n* (%)			0.140[1]
South Korea	7819 (96.7)	1152 (96.2)	
Vietnam	91 (1.1)	24 (2.0)	
China	99 (1.2)	11 (0.9)	
Philippines	25 (0.3)	6 (0.5)	
Japan	11 (0.1)	0	
Cambodia	17 (0.2)	1 (0.1)	
America	6 (0.1)	2 (0.2)	
Thailand	5 (0.1)	1 (0.1)	
Mongolia	10 (0.1)	1 (0.1)	

[1]Chi-squared test; [2]Student`s t-test; $P < 0.05$ is shown in bold.
