# Supplementary material for: Machine Learning Models for Predicting Mortality in 7472 Very Low Birth Weight Infants Using Data from a Nationwide Neonatal Network
Source: Diagnostics (Basel). 2022 Mar 3;12(3):625. doi: 10.3390/diagnostics12030625 (PMC8947011; doi:10.3390/diagnostics12030625)
Supplement: Supplementary file 1 [file diagnostics-12-00625-s001.zip › SupplementaryT_1B.pdf]

Supplementary Table S1B. Paternal social history between the Alive and the Death groups

Characteristics	Alive (n=8127)	Death (n=1207)	*P*-value
**Social History**			
Education, graduation, *n* (%)			0.395[1]
Elementary school	6 (0.1)	0	
Middle school	44 (1.0)	3 (0.5)	
High school	1009 (21.9)	147 (23.6)	
College or above	3555 (77.0)	474 (76.0)	
Nationality, *n* (%)			0.875[1]
South Korea	7957 (98.8)	1172 (98.8)	
China	64 (0.8)	8 (0.7)	
Japan	4 (0.1)	1 (0.1)	
America	15 (0.2)	3 (0.3)	
Canada	1 (0)	0	
Australia	4 (0.1)	2 (0.2)	
England	2 (0)	0	
Pakistan	2 (0)	0	
Germany	1 (0)	0	

[1]Chi-squared test; [2]Student`s t-test; $P < 0.05$ is shown in bold.
