# Supplementary material for: Machine Learning Models for Predicting Mortality in 7472 Very Low Birth Weight Infants Using Data from a Nationwide Neonatal Network
Source: Diagnostics (Basel). 2022 Mar 3;12(3):625. doi: 10.3390/diagnostics12030625 (PMC8947011; doi:10.3390/diagnostics12030625)
Supplement: Supplementary file 1 [file diagnostics-12-00625-s001.zip › SupplementaryT_2.pdf]

Supplementary Table S2. Comparison of variables between KNN registry and this study

Variables of KNN registry	Variables of this study
**Neonatal factors**	**Neonatal factors**
Male sex	Male sex
Gestational age	Gestational age
Body weight	.
1 min Apgar scores	.
5 min Apgar scores	5 min Apgar scores
Body temperature	Body temperature
Multiple births	.
Resuscitation at birth	Resuscitation at birth
**Maternal factors**	**Maternal factors**
Age, year	.
Diabetes mellitus	Diabetes mellitus
Hypertension	Hypertension
Chorioamnionitis	Chorioamnionitis
PROM	PROM
Antenatal steroid	Antenatal steroid
Cesarean delivery	Cesarean delivery
**Maternal social history**	
Education	
Marital status	
Nationality	
**Paternal social history**	
Education	
Ethnicity	

PROM = premature rupture of membranes
